# Impact of COVID-19 pandemic on the incidence and severity of myasthenia gravis in Korea: using National Health Insurance Service database

**DOI:** 10.3389/fneur.2024.1374370

**Published:** 2024-05-06

**Authors:** Sooyoung Kim, Eun Kyoung Lee, Hasung Kim, Hoseob Kim, Eunhee Sohn

**Affiliations:** ^1^Department of Neurology, Chungnam National University Hospital, Chungnam National University College of Medicine, Daejeon, Republic of Korea; ^2^Department of Neurology, Chungnam National University Sejong Hospital, Chungnam National University College of Medicine, Sejong, Republic of Korea; ^3^Data Science Team, Hanmi Pharm. Co., Ltd, Seoul, Republic of Korea

**Keywords:** coronavirus disease 2019, epidemiology, incidence, myasthenia gravis, severity

## Abstract

**Background:**

This study investigated the impact of COVID-19 pandemic on the incidence and severity of myasthenia gravis (MG) using the National Health Insurance Service (NHIS) database in Korea.

**Methods:**

We analyzed data from patients with MG in the NHIS registry from 2015 to 2021. MG was defined as (1) patients aged ≥18 years with the G70.0 code, and (2) patients who visited tertiary hospitals regarldless of department in Korea (outpatient clinics at least twice or hospitalization at least once), and (3) patients who were prescribed pyridostigmine as MG medications at least once. We designated pre-COVID-19 as 2019 and post-COVID-19 as 2021 and analyzed the MG incidence and prevalence in 2019 and 2021. We compared the clinical data of patients with MG between the two years. MG exacerbation was defined as the administration of intravenous immunoglobulin or plasma exchange. Analysis of COVID-19 cases was conducted using an integrated database from the Korea Disease Control and Prevention Agency and NHIS. Patients with MG were divided into two groups according to COVID-19 status to compare their clinical characteristics.

**Results:**

A total of 6,888 and 7,439 MG cases were identified in 2019 and 2021, respectively. The standardized incidence was 1.56/100,000 in 2019, decreasing to 1.21/100,000 in 2021. Although the frequency of MG exacerbations was higher in 2019, there were no differences in the number and duration of hospitalizations, duration of ICU stays, hostalization expense, and mortality between 2019 and 2021. Patients with MG and COVID-19 had a higher frequency of MG exacerbations than patients without COVID-19, but there were no differences in the number and duration of hospitalizations, hospitalization expense, and mortality.

**Conclusion:**

This study was the first nationwide population-based epidemiological study of MG during COVID-19 pandemic in Korea. The incidence of MG decreased during COVID-19 pandemic, and the severity of MG was not affected by COVID-19.

## Introduction

Coronavirus disease 2019 (COVID-19) is a global pandemic caused by the severe acute respiratory syndrome coronavirus 2 (SARS-CoV-2), and SARS-CoV-2 continues to mutate and produce variants, causing repeated infections. There are several concerns regarding the COVID-19 pandemic in patients with myasthenia gravis (MG), including vulnerability to COVID-19 infection, as many patients have been administered immunosuppressive agents, and aggravation of MG symptoms such as fatigability or weakness of the respiratory muscles resulting from COVID-19 infection. Additionally, drugs such as hydroxychloroquine and azithromycin, which are used to treat COVID-19 infection, can worsen the symptoms of MG (1, 2). To date, previous studies and case series have reported the impact of COVID-19 on MG. However, most of these studies were conducted on a limited number of cases and for a limited period. Although some reported cases of new-onset MG have described a possible connection between SARS-CoV-2 infection and MG occurrence (3–7), there have been few nationwide population-based research on the relationship between COVID-19 pandemic and the incidence of MG to date. A previous study in Germany reported in 2023 conducted a nationwide assessment of the incidence of MG in COVID-19 pandemic. In this study, the age-adjusted mean incidence rate weas 2.8 per 100,000 people-years between 2011 and 2020, and decreased significantly in 2020 (8). A cross-sectional study by Camelo et al. reported that of patients with COVID-19 and MG, 87% were admitted to the intensive care unit (ICU), 73% required mechanical ventilation, and 30% died (9). Conversely, Actoz and colleagues reported that most patients with MG showed good recovery from COVID-19, and those with high MG Activity of Daily Living (MG-ADL) scores prior to the COVID-19 pandemic and those with a history of MG crises were at higher risk of developing severe COVID-19 (10). Controversy surrounds the severity of MG during the COVID-19 pandemic.

An epidemiological study of the relationship between COVID-19 and MG has not yet been performed in Korea. Therefore, this study investigated the impact of COVID-19 pandemic on the incidence and severity of MG using the National Health Insurance Service (NHIS) database in Korea.

## Materials and methods

### Data source and selection of study population

Using the NHIS database, claims data on diagnosis, comorbidities, procedures, prescription records, healthcare facility visit records, and demographic information were obtained, and data from January 1, 2015, to December 31, 2021, were retrospectively reviewed. The NHIS is a universal health management system that covers 98% of the Korean population (11). The case of MG was defined as patients aged ≥18 years with the G70.0 code designated as MG in the International Classification of Disease, 10th revision, and to increase the diagnostic accuracy, and we added the followings: (a) patients who visited tertiary hospitals regardless of department in Korea (outpatient clinics at least twice or hospitalization at least once), and (b) patients who were prescribed pyridostigmine as MG medications at least once.

### Estimation of incidence and prevalence

We designated pre-COVID-19 as 2019 and post-COVID-19 as 2021 and analyzed the incidence and prevalence of MG in 2019 and 2021. Since 2020 was before the outbreak of the COVID-19 pandemic in Korea, we excluded this period from the post-COVID-19 designation. We calculated the prevalence and incidence from 2017 to 2021. To calculate the incidence, a “wash-out” period of two years before each year was designated. Incident cases were analyzed according to age and sex. In addition, to eliminate effects of age and sex structure of cohort, we calculated by utilizing indirect method such as the standardized incidence rate and prevalence rate using the standard population data for 2021 from the Korean Statistical Information Service of the Korean National Statistical Office. In specific, the standardized incidence rate was estimated by dividing the number of observed incidence cases by the number of expected incidence cases. The standardized prevalence rate was also calculated, in a similarly method.

### Comparison of clinical data between the patients with MG in pre-COVID-19 and post-COVID-19

We compared the demographic and clinical data of patients with MG and patients with MG exacerbation between 2019 and 2021. Clinical variables, including age, sex, comorbidities, history of thymectomy, the numbers of prescribed MG medication including immunosuppressive agents, presence of MG exacerbation, record of hospitalization, ICU admission, annual hospitalization expense, and mortality, were collected. A MG exacerbation was defined as the administration of intravenous immunoglobulin (IVIg) or plasma exchange. NHIS only searches for benefit administration of IVIg. The maintenance treatment of IVIg is non-benefit administration in Korea, so patients receiving maintenance treatment were not included in the analysis. The severity of MG was evaluated based on the presence of MG exacerbations, the average number and duration of hospitalizations, including ICU admissions, hospitalization expense, and mortality.

### Analysis of COVID-19 confirmed cases

COVID-19-confirmed cases were analyzed using an integrated database from the Korea Disease Control and Prevention Agency (KDCA) and NHIS. COVID-19-confirmed cases were defined as patients with positivity for COVID-19 polymerase chain reaction test result. Based on the data on COVID-19 cases, patients with MG were divided into two groups according to COVID-19 infection status to compare their clinical characteristics and MG severity. To this analysis, clinical variables, including age, sex, comorbidities, history of thymectomy, presence of MG exacerbations, record of hospitalization, ICU admission, annual hospitalization expense, and mortality, were collected.

### Statistical analysis

A chi-square and student *t*-test were performed to compare the statistical significance of data in 2019 and 2021 and data from the two groups according to COVID-19 infection status. All statistical analyses were performed using SAS version 9.4 (SAS Institute, Cary, NC, USA). Statistics for the clinical variables are presented as proportions, means, standard deviations, and ranges. Statistical significance was set at a *p* value less than 0.05. The map figures in this study were creased by utilizing ArcGIS software from ESRI (Redlands, CA).

## Results

### Incidence and prevalence of MG

**Figure 1:**
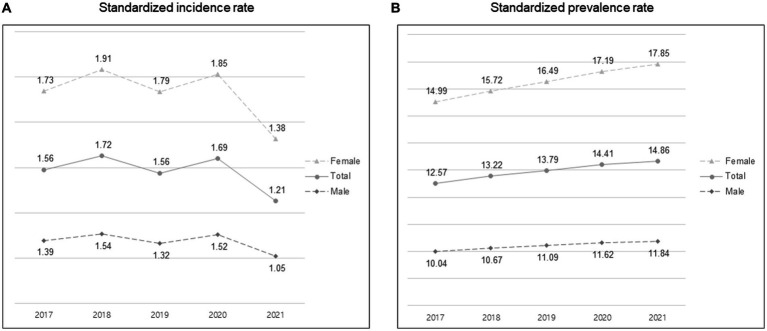
Standardized incidence and prevalence rates per 100,000 person-years according to sex from 2017 to 2021. **(A)** Standardized incidence rate. **(B)** Standardized prevalence rate.

**Figure 2:**
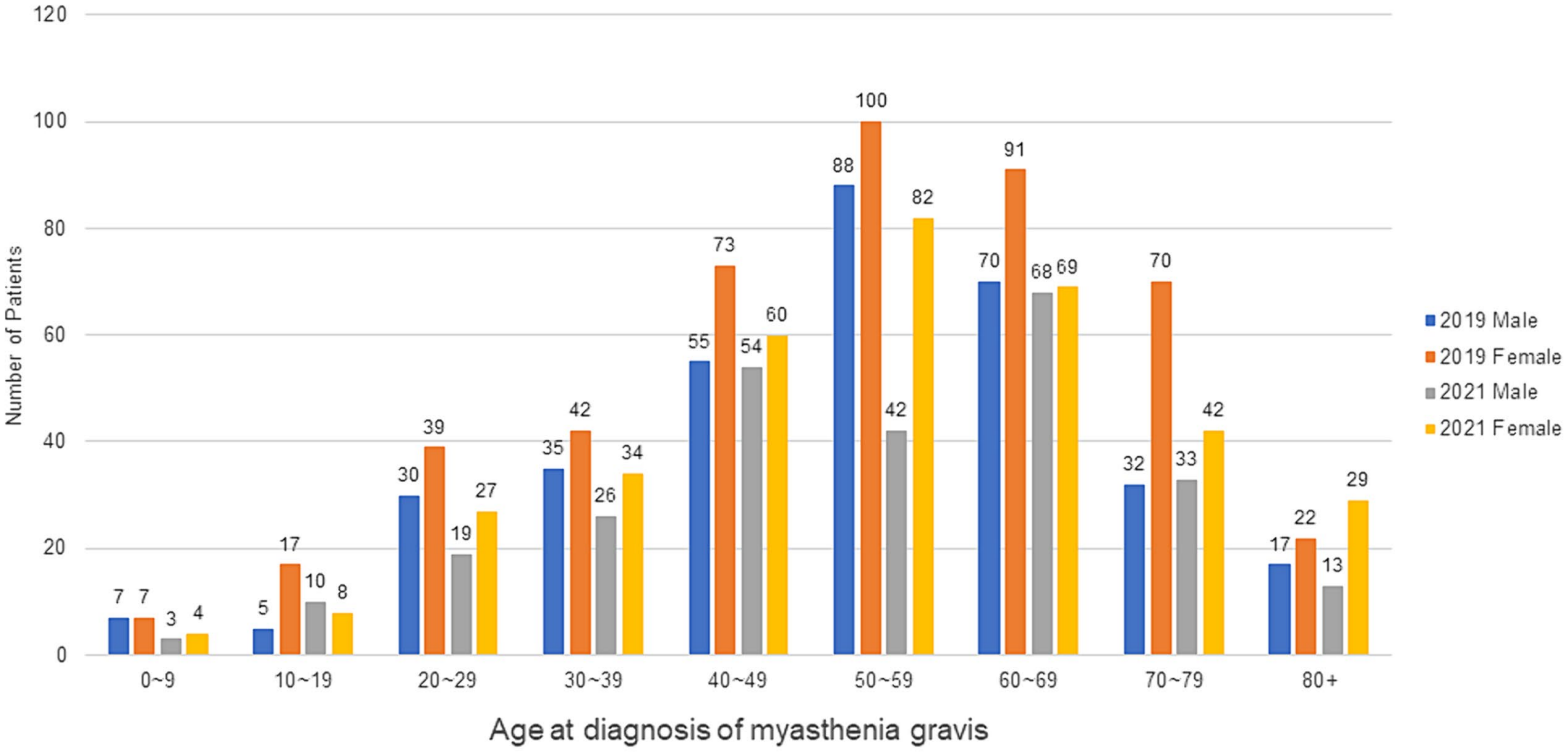
Incident cases of myasthenia gravis according to age groups and sex in 2019 and 2021.

**Figure 3:**
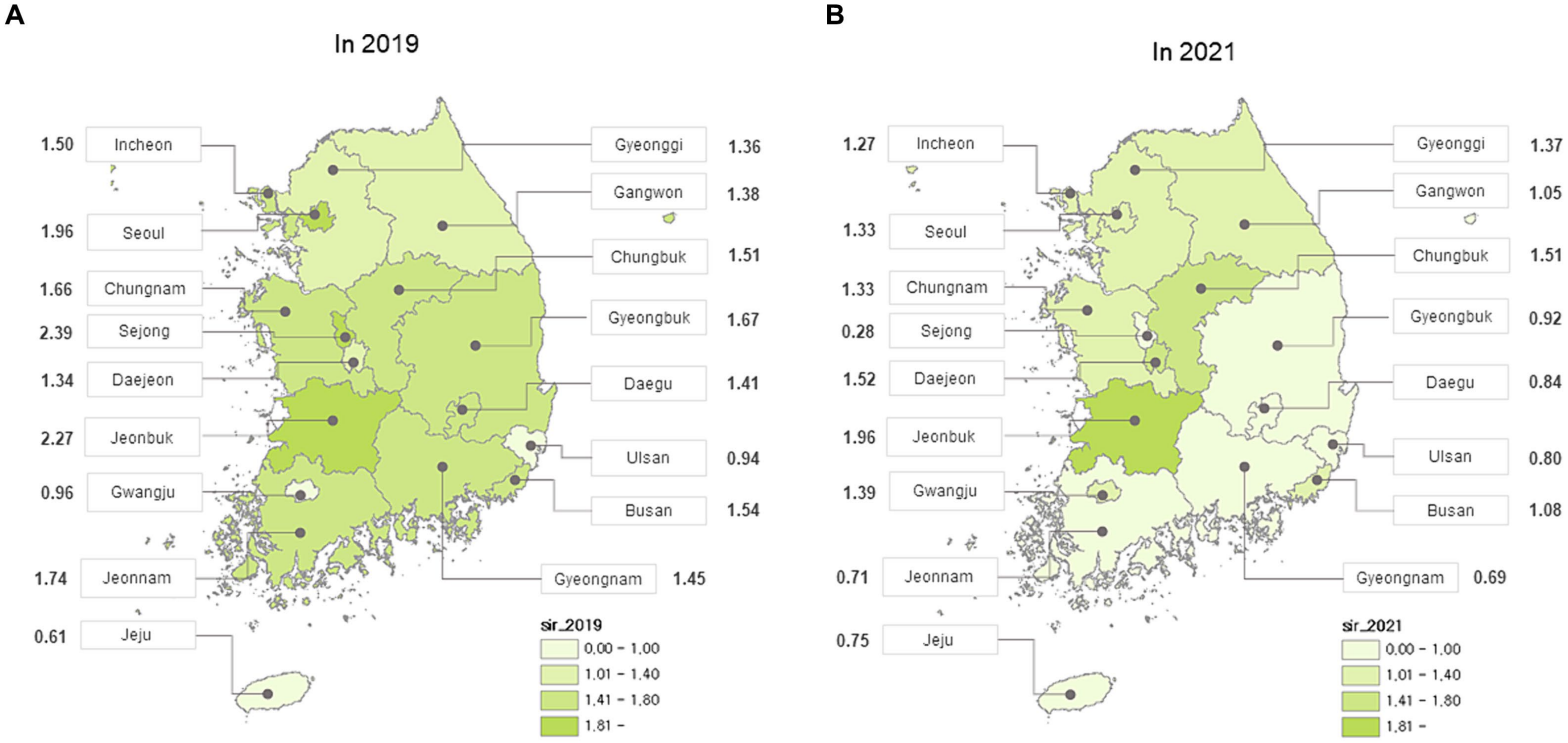
Standardized incidence rate per 100,000 person-years according to region that included 17 cities and provinces from Korea **(A)** in 2019 **(B)** in 2021.

In total, 6,888 MG cases (male: 2,770; female: 4,118) were identified in 2019, and 7,439 MG cases (male: 2,962; female: 4,477) in 2021. The standardized incidence was 1.56/100,000 in 2019, decreasing to 1.21/100,000 in 2021. The standardized incidence and prevalence rates per 100,000 person-years according to sex from 2017 to 2021 are shown in Figure 1. The number of MG occurrence according to age group and sex in 2019 and 2021 is shown in Figure 2. The incident cases tended to be female patients, accounting for a larger proportion than males in each age group, and showed a peak from 50–59 years old in 2019 to 60–69 years old in 2021. Overall, there were more incident cases in 2019 than in 2021. The standardized incidence rates per 100,000 person-years according to the 17 cities and provinces in Korea in 2019 and 2021 are presented in Figure 3. The regions where standardized incidences decreased the most in 2021 were as follows in order: Sejong (2.39 → 0.28), Jeonnam (1.74 → 0.71), Gyeongnam (1.45 → 0.69), Gyeongbuk (1.67 → 0.92), and Seoul (1.96 → 1.33).

### Comparisons of clinical data of the patients with MG between 2019 and 2021

The baseline characteristics of the patients with MG in 2019 and 2021 are shown in Table 1. The mean age at first diagnosis was 56.1 ± 15.6 years in 2019 and 57.3 ± 15.6 years in 2021, with a statistically significant difference (^***^p < 0.0001). In addition, the presence of hypertension was higher in patients with MG in 2021 than in 2019 (hypertension: 31.0% in 2019 and 33.6% in 2021, ^***^p = 0.0009). There were no differences in comorbidities between 2019 and 2021 excluding hypertension, history of thymectomy, number of immunosuppressants, and mortality. The frequency of MG exacerbations was significantly higher in 2019, with 206 cases in 2019 and 178 cases in 2021 (^*^p = 0.03).

**Table 1:** Baseline characteristics between patients with myasthenia gravis (MG) in 2019 and 2021.

Variables	MG in 2019		MG in 2021		*P* value
	*n* = 6,888	100 (%)	*n* = 7,439	100 (%)	
Age (years)	56.1 ± 15.6		57.3 ± 15.6		<0.0001^***^
Men [*n* (%)]	2,770	40.2	2,962	39.8	0.63
Comorbidities [*n* (%)]					
Diabetes	1,548	22.5	1,754	23.6	0.12
Hypertension	2,133	31.0	2,497	33.6	0.0009^***^
Autoimmune disease					
RA	49	0.7	61	0.8	0.46
SLE	36	0.5	52	0.7	0.18
SjS	47	0.7	43	0.6	0.43
AS	29	0.4	33	0.4	0.84
Hashimoto’s thyroiditis	108	1.6	130	1.8	0.40
Thymectomy [*n* (%)]	27	0.39	43	0.58	0.11
IS agent [*n* (%)]					0.17
None	2,746	40.0	2,859	38.4	
1 IS group	2,444	35.5	2,668	35.9	
≥ 2 IS group	1,698	24.7	1,912	25.7	
MG exacerbation [*n* (%)]	206	3.0	178	2.4	0.03^*^
Mortality [*n* (%)]	84	1.2	104	1.4	0.3

MG, myasthenia gravis; RA, rheumatoid arthritis; SLE, systemic lupus erythematosus; SjS, Sjögren’s syndrome; AS, ankylosing spondylitis; IS agent, immunosuppressive agent. ^*^Indicates that the results are statistically significant (*p* < 0.05), ^***^indicates that the results are statistically significant (*p* < 0.001).

Comparisons of the clinical data of patients with MG exacerbations between 2019 and 2021 are presented in Table 2. Although the frequency of MG exacerbation was higher in 2019, there were no differences in the number and duration of hospitalizations, the duration of ICU stays, hospitalization expense and mortality between 2019 and 2021 among patients with MG exacerbations. The history of thymectomy increased significantly in 2021 (^*^p = 0.01).

**Table 2:** Baseline characteristics between patients with myasthenia gravis (MG) exacerbation in 2019 and 2021.

Variables	MG exacerbation in 2019		MG exacerbation in 2021		*P* value
	*n* = 206	100 (%)	*n* = 178	100 (%)	
Age (years)	54.7 ± 16.1		55.0 ± 17.1		0.88
Men [*n* (%)]	77	37.4	62	34.8	0.60
Comorbidities [*n* (%)]					
Diabetes	45	21.8	42	23.6	0.68
Hypertension	73	35.4	55	30.9	0.35
Autoimmune disease					
RA	2	1.0	2	1.1	0.88
SLE	0	0.0	0	0.0	NA
SjS	2	1.0	2	1.1	0.88
AS	2	1.0	2	1.1	0.88
Hashimoto’s thyroiditis	5	2.4	5	2.8	0.81
Thymectomy [*n* (%)]	27	13.1	43	24.2	0.01^*^
Frequency of chest CT performance	170	82.5	142	79.8	0.49
Exacerbation management [*n* (%)]					
IV immunoglobulin	122	59.2	92	51.7	0.14
Plasma exchange	149	72.3	126	70.8	0.74
ICU stay duration (days)	26.2 ± 24.1		24.1 ± 23.6		0.5
The average number of ICU admission	1.3 ± 0.7		1.2 ± 0.5		0.5
Hospitalization duration (days)	46.8 ± 63.7		35.6 ± 52.9		0.07
The average number of hospitalizations	3.0 ± 3.1		2.6 ± 2.8		0.15
Hospitalization expense (million won)	27.3 ± 30.0		29.2 ± 31.1		0.56
Mortality [*n* (%)]	22	10.7	16	9.0	0.6

MG, myasthenia gravis; RA, rheumatoid arthritis; SLE, systemic lupus erythematosus; SjS, Sjögren’s syndrome; AS, ankylosing spondylitis; CT, computed tomography; IV, intravenous; ICU, intensive care unit. NA indicates that the result cannot be generated, ^*^indicates that the results are statistically significant (*p* < 0.05).

### Analysis of COVID-19 confirmed cases

Patients with MG and COVID-19 infection had a higher frequency of MG exacerbations (^**^p = 0.008) than patients with MG without COVID-19, but there were no differences in the number and duration of hospitalizations, hospitalization expense, and mortality (Table 3).

**Table 3:** Baseline characteristics between patients with myasthenia gravis (MG) and COVID-19 infection and those with MG without COVID-19 infection in 2021.

Variables	With COVID-19 in MG		Without COVID-19 in MG		*P* value
	*n* = 90	100 (%)	*n* = 7,349	100 (%)	
Age (years)	57.3 ± 15.6		55.6 ± 14.1		0.31
Men [*n* (%)]	38	42.2	2,924	39.8	0.64
Comorbidities [*n* (%)]					
Diabetes	20	22.2	1,734	23.6	0.76
Hypertension	32	35.6	2,465	33.5	0.69
Autoimmune disease					
RA	1	1.1	60	0.8	0.76
SLE	1	1.1	51	0.7	0.64
SjS	1	1.1	42	0.6	0.50
AS	0	0.0	33	0.5	0.52
Hashimoto’s thyroiditis	1	1.1	129	1.8	0.64
Thymectomy [*n* (%)]	1	1.1	43	0.6	0.50
MG exacerbation [*n* (%)]	6	6.7	172	2.3	0.008^**^
ICU stay duration (days)	17.0 ± 10.4		24.4 ± 23.9		0.60
The average number of ICU admission	1.0 ± 0.0		1.2 ± 0.5		0.0003^***^
Hospitalization duration (days)	27.3 ± 35.2		35.9 ± 53.5		0.70
The average number of hospitalizations	1.8 ± 1.2		2.6 ± 2.8		0.50
Hospitalization expense (million won)	6.8 ± 9.4		8.3 ± 13.4		0.64
Mortality [*n* (%)]	2	2.2	102	1.4	0.50

COVID-19, coronavirus disease 2019; MG, myasthenia gravis; RA, rheumatoid arthritis; SLE, systemic lupus erythematosus; SjS, Sjögren’s syndrome; AS, ankylosing spondylitis; ICU, intensive care unit ^**^indicates that the results are statistically significant (*p* < 0.01), ^***^indicates that the results are statistically significant (*p* < 0.001).

## Discussion

In this nationwide population-based epidemiological study, the incidence and severity of MG, defined as the presence of MG exacerbation, numbers and duration of hospitalization, annual hospitalization expense, and mortality, did not increase after the COVID-19 pandemic. Although the frequency of MG exacerbation was higher in 2019, there were no differences in the number and duration of hospitalizations, the duration of ICU stays, hospitalization expense and mortality between 2019 and 2021. In addition, MG patients with COVID-19 infection had higher frequency of MG exacerbation than MG patients without COVID-19 infection, but there were no differences in number and duration of hospitalization, hospitalization expense and mortality.

This study showed that the standardized incidence of MG in Korea was 1.56/100,000 in 2019, decreasing to 1.21/100,000 in 2021. In Korea, many healthcare centers were converted to medical centers dedicated to COVID-19 to prevent the spread of COVID-19 in 2021 based on national policy, as the outbreak of COVID-19 started in Korea after August 2020 and severe cases of COVID-19 infections have increased a lot since 2021, which seemed to have led to a delayed diagnosis of MG. Furthermore, it is assumed that many patients with mild symptoms of MG avoided seeking medical attention due to their reluctance to engage in outdoor activities and healthcare center visits, which peaked during this time. In addition, activities such as social isolation and restrictions on going out were encouraged to prevent the spread of infection in national administrative aspects. According to a medical claims data-based study published in Germany in 2023, the age-adjusted mean incidence rate was 2.8 per 100,000 person-years between 2011 and 2020 and decreased significantly in 2020, coinciding with the start of the COVID-19 pandemic. Similarly, the hospitalization rate of patients with MG decreased to an overall low rate of 8.1% in 2020. This decrease was attributed to a disruption to the usual healthcare system in Germany at the beginning of 2020 (8). However, there is still a lack of research on the nationwide population-based incidence rate of MG during the COVID-19 pandemic in many countries.

In this study, we observed that the frequency of MG exacerbations was higher in 2019 than in 2021. The lower number of patients with MG exacerbations in 2021 compared to 2019 may be attributed to the same factors contributing to the decrease in incidence rates during the COVID-19 pandemic.

The proportion of thymectomy in patients with MG exacerbation increased during the post-COVID period. Since there was no difference in the frequency of chest imaging between patients with MG exacerbation in 2019 and 2021 (170/206 in 2019, 142/178 in 2021, *p* = 0.49), the increase of thymectomy during the post-COVID period is independent of the frequency of chest imaging. We suggested that thymectomy has been actively encouraged in the 2020 American academy of neurology guideline for MG and have an impact on this. Although surgical activity decreased worldwide during the COVID-19 pandemic, the number of elective and emergency operations increased in Korea in 2021. In 2020, it decreased slightly compared to the previous year, but rather, in 2021, the overall number of operations including thymectomy increased. This suggests that the incidence rate decreased by switching to healthcare centers to dedicated COVID-19, but surgery for previous diagnosed diseases proceeded as planned, if necessary, in Korea.

We also found that patients with MG and COVID-19 infection had a higher frequency of MG exacerbations than patients with MG without COVID-19 infection; however, there were no differences in the number and duration of hospitalizations, hospitalization expense and mortality, suggesting that the severity of MG was not affected by COVID-19. Although several previous studies show that COVID-19 is related to the aggravation of MG and an increase in its mortality (12–17), most of these are data from limited populations and period-based research. In 2023, there was a relatively large-scale study of residents living in Ontario, Canada, and the study compared hospitalization and mortality rates in COVID-19 contracted patients with MG, patients with rheumatoid arthritis (RA), and general population, respectively (17). Compared with general population controls and controls with RA, patients with MG had higher rate of COVID-19 associated emergency department visits, hospitalization admissions, and 30-day mortality in previous study. However, in general, because patients with MG are more likely to worsen to infection, especially respiratory infection such as COVID-19 than patients with RA or general population, it would be more meaningful to assess the impact of COVID-19 infection on severity of MG within a disease group than to compare between various disease groups.

On the other hands, the French database-based cohort study showed that COVID-19 had a limited effect on most patients with MG, and immunosuppressive medications and corticosteroids used for MG treatment were not related to poor outcomes in patients with COVID-19 (18). A study examining patients with multiple sclerosis (MS) and the general population with confirmed COVID-19 reported that COVID-19 did not cause significant harm or increase mortality in patients with MS. Moreover, the use of disease-modifying therapies did not appear as a significant risk factor for poor COVID-19 outcomes; however, there is a possibility that the usage of B-cell-depleting therapies could exacerbate COVID-19 infections (19). While the hypothesis that COVID-19 may have less impact on the aggravation of MG remains uncertain, increasing evidence suggests that immunosuppression may have protective effects through immune response limitation (20).

This study had several limitations. First, COVID-19 continues to spread, and its variants are evolving. However, since this study defined only 2021 as the post-COVID-19 pandemic period, it does not accurately reflect the constantly changing situation. In Korea, COVID-19 vaccination commenced since February 2021 and the serial vaccinations have been implemented until now. In addition, the Omicron variant has appeared in Korea since January 2022. However, our study was not included the information on COVID-19 vaccination and the situation after the emergence of the Omicron variant. Especially, the decrease in the incidence of MG in 2021, only an year of beginning COVID-19 pandemic, seems to reflect more the aspect of social factors than pathophysiological factors of MG. More acute results could have been obtained if the analysis period of post COVID-19 pandemic was long. Second, the NHIS database does not include medical records from physicians, such as the MG-ADL, Myasthenia Gravis Foundation of America classification, and quantitative myasthenia gravis score. Therefore, symptom severity could not be assessed in patients with mild symptom exacerbations who were not treated with IVIg or plasma exchange and did not receive treatment at healthcare centers. Third, when MG and COVID-19 co-occur, their temporal causal relationship is unclear. Fourth, patients could not be analyzed according to the presence of MG-specific antibodies due to the nature of NHIS data analysis. The lack of information on the serological status of patients is a limitation because the presence of specific antibodies is an important factor defining the patient population and influencing the treatment used. Despite several limitations, the main strength of this study is the large size of the analytical cohort based on a nationwide population database. These novel data allowed us to assess the impact of COVID-19 on the incidence and severity of MG with sufficient statistical power.

In conclusion, this is the first nationwide population-based epidemiological study of MG during the COVID-19 pandemic in Korea. Based on the data from the NHIS database, from 2015 to 2021, the incidence of MG decreased during the COVID-19 pandemic, and there was no significant change the severity of MG due to COVID-19. We expect this study to contribute to further epidemiological studies on the relationship between MG and COVID-19.

## Data availability statement

The original contributions presented in the study are included in the article/supplementary material, further inquiries can be directed to the corresponding author.

## Ethics statement

The studies involving humans were approved by the Institutional Review Board of the Chungnam National University Hospital (IRB number: 2022-05-037). The studies were conducted in accordance with the local legislation and institutional requirements. Written informed consent for participation was not required from the participants or the participants’ legal guardians/next of kin in accordance with the national legislation and institutional requirements.

## Author contributions

SK: Conceptualization, Methodology, Writing – original draft, Writing – review & editing. EKL: Conceptualization, Methodology, Writing – review & editing. HaK: Data curation, Investigation, Writing – review & editing. HoK: Data curation, Formal analysis, Investigation, Methodology, Writing – review & editing. ES: Conceptualization, Funding acquisition, Methodology, Supervision, Writing – review & editing.
